# Comparison of the efficacy and safety of nafamostat mesylate and heparin in blood purification therapy for patients with severe acute pancreatitis: protocol for a randomized controlled trial

**DOI:** 10.3389/fmed.2026.1868927

**Published:** 2026-08-03

**Authors:** Hui Wang, Xiang Gao, Chunfeng Lu, Alin Sun, Qinghai Zhang, Zhipeng Gao

**Affiliations:** 1 Department of Critical Care Medicine, Hangzhou Lin’an Traditional Chinese Medicine Hospital, Hangzhou, Zhejiang, China; 2 Department of Critical Care Medicine, Weifang People’s Hospital, Weifang, Shandong, China

**Keywords:** continuous renal replacement therapy, heparin, nafamostat, pancreatitis, safety

## Abstract

**Background:**

Blood purification plays an important role in the treatment of severe acute pancreatitis. Extracorporeal blood anticoagulation is an important strategy to achieve smooth circulation in blood purification circuits. We aimed to investigate the efficacy, safety, and impact on circulating cytokines of using heparin or nafamostat mesylate anticoagulation in patients with severe acute pancreatitis during continuous renal replacement therapy.

**Methods and analysis:**

This is a single-center randomized controlled study. We plan to recruit adult patients with severe acute pancreatitis from intensive care units for this study. Participants who met the criteria were randomly assigned in a 1:1 ratio, with one group using heparin anticoagulation during continuous renal replacement therapy and the other group using nafamostat mesylate anticoagulation. The primary outcome measure was mean filter life. Secondary outcome measures included changes in Sequential Organ Failure Assessment (SOFA), the Acute Physiology and Chronic Health Evaluation II (APACHE II) score, and inflammatory factors (CRP, PCT, IL-6, IL-8, IL-10, TNF-α) compared with baseline levels on days 1, 3, 5, and 7 after treatment, time to successful initiation of enteral nutrition, blood transfusion requirements, ICU length of stay and total hospitalization days, and follow-up of patients’ blood purification dependency rate and all-cause mortality at 28 days. Safety indicators include the occurrence of any bleeding events (defined by ISTH criteria), filter clotting, and the incidence of heparin-induced thrombocytopenia.

**Discussion:**

The results of this study are expected to provide guidance for the selection of anticoagulant drugs for critically ill patients with severe acute pancreatitis who receive continuous renal replacement Therapy, and to clarify the optimal anticoagulation strategy for this population, thereby optimizing clinical practice for treating SAP patients.

**Clinical trial registration:**

[https://clinicaltrials.gov/], identifier [ChiCTR2500 111161]

## Introduction

### Background and rationale

Severe acute pancreatitis (SAP) is a common type of critical abdominal condition in clinical practice. Due to its rapid progression, SAP frequently prone to cause multiple organ dysfunction or even failure, resulting in high complication and mortality rates (1, 2). In patients with SAP who develop persistent organ failure, the mortality rate can be as high as 40% (3–5). Due to the changes in the body functions and circulation of patients with severe acute pancreatitis, the levels of inflammatory factors in their bodies will show a significant upward trend, thereby triggering a cascading reaction of inflammatory factors and subsequently leading to multiple organ dysfunction (6, 7). Previous studies (1, 6–8) have shown that in the early phase of SAP, the excessive release of inflammatory factors will further aggravate the severity of systemic inflammatory response syndrome and promotes the development of infected pancreatic necrosis. Therefore, inflammatory factors are significant factors influencing the onset and treatment of patients with SAP, and they are also important indicators reflecting the condition of patients with SAP (1, 9, 10).

Blood purification therapy can help restore the immune homeostasis of patients with SAP by removing various inflammatory mediators, inhibiting the cascade of inflammatory reactions, and modulating the immune responses. This approach may prevent the development of various severe complications in SAP patients and improve the clinical outcomes (11–13). Consequently, continuous renal replacement therapy has become one of the important treatment methods for SAP. In continuous renal replacement therapy, effective anticoagulation is essential for ensuring the smooth operation of the filter and achieving the target dialysis dose. Heparin has become a commonly used anticoagulant in clinical practice due to its clear anticoagulant effect and easy monitoring. Moreover, studies have shown that heparin can improve pancreatic microcirculation and mitigate pancreatic tissue damage (14–16). However, heparin may cause bleeding and heparin-induced thrombocytopenia (HIT). Some patients still experience recurrent filter clotting and leading to interruption of continuous renal replacement therapy, even while receiving heparin anticoagulation (17). Furthermore, for patients with clotting disorders or those at risk of bleeding, the use of heparin is usually restricted. Nafamostat mesylate (NM) is a synthetic serine protease inhibitor that can exert anticoagulant effects by directly inhibiting coagulation factors, regulating fibrinolysis, and modulating platelet function (18). In addition, some studies (19–21) have shown that NM can suppress trypsin activity and enhance pancreatic microcirculation, which might be helpful for the treatment of severe acute pancreatitis (SAP). To date, no studies have systematically evaluated or compared the clinical efficacy and safety of nafamostat mesylate versus heparin as anticoagulants in patients with severe acute pancreatitis undergoing continuous renal replacement therapy.

We conducted a randomized controlled trial to compare the efficacy and safety of nafamostat mesylate and heparin as anticoagulants during CRRT in patients with severe acute pancreatitis. The results of this study may provide evidence-based medicine for optimizing anticoagulation strategies in this special population, which will help guide clinical practice and ultimately improve the clinical prognosis of SAP patients.

### Objectives

This study primarily compared the effects of nafamostat mesylate and heparin on the lifespan of blood filters during continuous renal replacement therapy in patients with severe acute pancreatitis. The secondary objective was to evaluate their differential impacts on organ dysfunction, inflammatory and coagulation responses, time to gastrointestinal function recovery, clinical outcomes, and overall safety.

### Trial design

**FIGURE 1:**
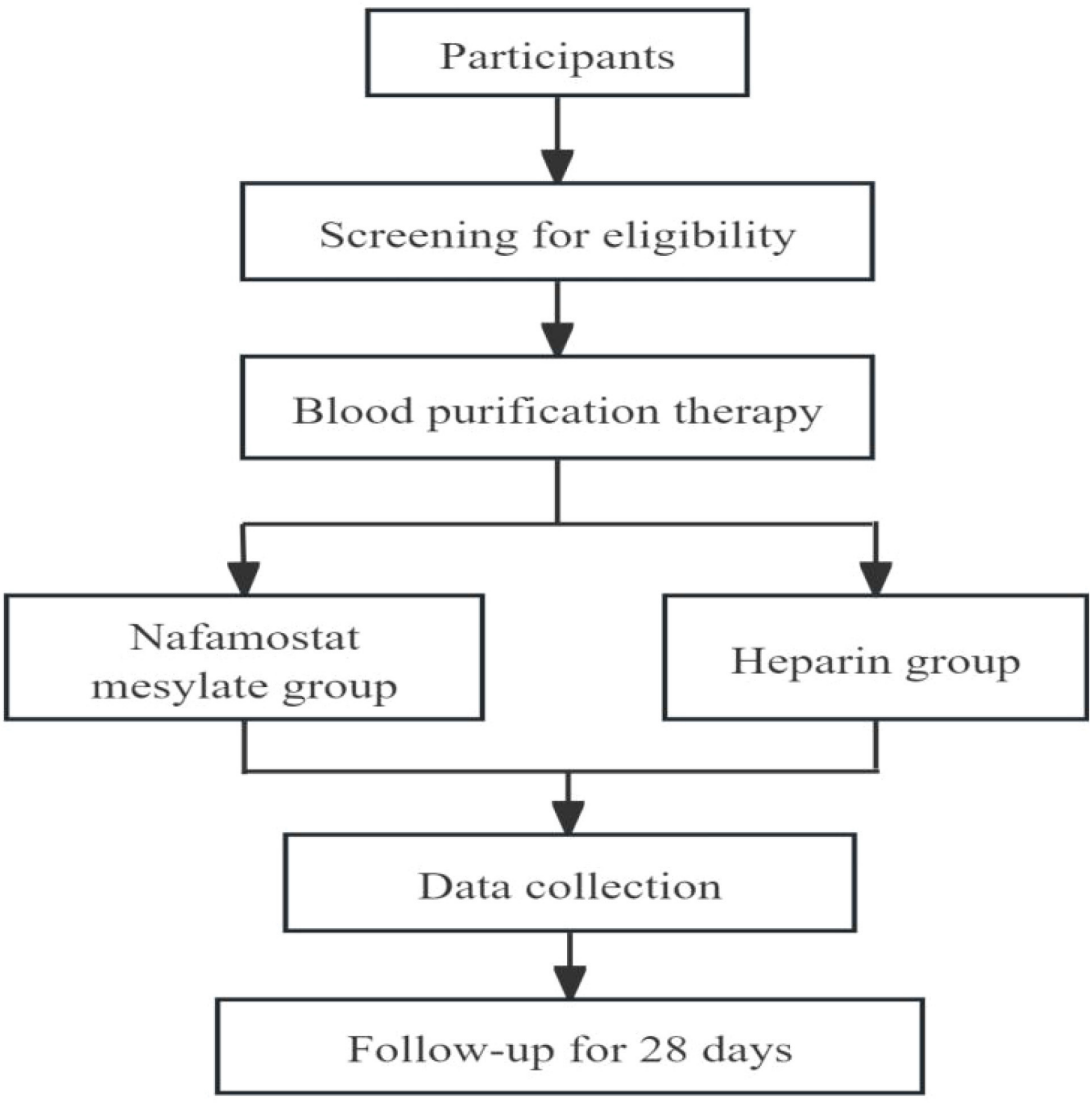
Flow chart of the research.

This study was designed as a prospective, single-center, randomized, controlled, open-label clinical trial, and reported in accordance with the SPIRIT 2013 reporting guidelines (22). Participants who meet the criteria will be randomly assigned in a 1:1 ratio to receive either NM or UFH as anticoagulants during the CRRT process. Figure 1 shows the flow chart of the research.

## Methods: participants, interventions, and outcomes

### Study setting

This study will be conducted in the Department of Intensive Care Medicine of Weifang People’s Hospital, Weifang, Shandong Province, China. The intensive care unit has a total capacity of 110 beds.

### Eligibility criteria

Participants will be considered for inclusion in the study if they meet the following criteria upon admission to the ICU or during stay in the ICU: (1) adults over 18 years old, (2) diagnosed with severe acute pancreatitis (SAP) according to the revised Atlanta classification (2012), meeting at least one of the following: Persistent organ failure ( > 48 h), or APACHE II score ≥ 8 or SOFA score ≥ 4 at ICU admission, (3) requiring blood purification therapy [continuous renal replacement therapy (CRRT)] due to systemic inflammatory response, acute kidney injury, or hypercytokinemia, (4) informed consent obtained from patient or surrogate.

The exclusion criteria for participants included the following: (1) known hypersensitivity or contraindication to NM or UFH, (2) severe coagulation disorders (INR > 2.0), active major bleeding (such as intracranial hemorrhage, ongoing gastrointestinal bleeding) or recent uncontrolled bleeding and bleeding diseases or platelet count < 30 × 10^9^/L, (3) severe hepatic failure (Child–Pugh C), (4) History of heparin-induced thrombocytopenia (HIT), (5) pregnancy or lactation. (6) Chronic kidney disease requiring dialysis before ICU admission, (7) expected survival < 48 h or refusal to participate.

### Who will obtain informed consent or assent from potential trial participants or authorized surrogates?

Before patients participate in the research, the trained study investigators will explain the objectives, procedures, potential risks and benefits of the study, and answer any questions from each patient or their legal representative in detail. After they fully understand the research and voluntarily agreed to participate, they will sign a written informed consent form. Participation in this study is voluntary. Participants can withdraw from the study at any time without affecting their subsequent treatment.

### Additional consent provisions for collection and use of participant data and biological specimens

Not applicable. The data used in this trial will be obtained from the patients’ medical records generated during hospitalization. The researchers will collect only the information required for this study. To ensure participants’ privacy and data security, all information related to the patients’ identities will be anonymized. This study does not involve the collection, storage or future use of biological samples. Therefore, no supplementary consent form is required for the use of biological samples.

## Interventions

### Explanation for the choice of comparators

Heparin is a commonly used anticoagulant in blood purification therapy, and thus can be used as a suitable control. However, patients with SAP often have a higher risk of bleeding, which limits the use of heparin. Nafamostat mesylate can exert anticoagulant effects by inhibiting coagulation factors, and it metabolizes rapidly, having minimal effects on the entire body. Therefore, the application of nafamostat mesylate is becoming increasingly widespread. Comparing nafamostat with the current practice of heparin will help determine whether it provides safer or more reliable anticoagulation during CRRT in this population.

### Intervention description

The CRRT modality will be standardized as continuous venovenous hemodiafiltration (CVVHDF) throughout the trial. All patients will receive standardized CRRT delivered using the Gambro^®^ Prismaflex system, with a prescribed effluent dose of 20∼35 mL/kg/h. Within this prescribed range, the effluent dose and ultrafiltration rate will be titrated according to the patient’s hemodynamic stability (e.g., mean arterial pressure and vasopressor dose) and the severity of the systemic inflammatory response (e.g., procalcitonin, lactate, and SOFA score), consistent with current practice for CRRT in critically ill patients with hypercytokinemia. Vascular access will be obtained via a double-lumen catheter in the internal jugular or femoral vein.

The participants were randomly assigned to the nafamostat mesylate group or the heparin group. The patients randomly assigned to the NM group will only receive nafamostat mesylate as an anticoagulant during CRRT. Dissolve and prepare the NM injection solution using 5% glucose injection. The prepared nafamostat injection will be continuously injected through the anticoagulant injection tube at the same time as the start of extracorporeal circulation and the initial dose is generally 20–50 mg/h. During treatment, the dose is adjusted dynamically based on comprehensive considerations such as extracorporeal circulation, dialyzer usage, and bleeding risk. The general range of dose adjustment each time is 5–10 mg/h. Patients in the heparin group will only receive standard heparin anticoagulation treatment. At the beginning of treatment, 5–15 U/kg of heparin was injected intravenously for loading therapy. Heparin was then continuously infused through the anticoagulant injection tube at a rate of 5–10 U/kg/h. In case of significant bleeding or clotting events, dosage will be adjusted or anticoagulation discontinued according to protocol.

During the research period, the researchers will not attempt to change or influence other routine clinical treatments for patients with severe acute pancreatitis. The treatment plan will be determined by the clinical staff based on the relevant guidelines for severe acute pancreatitis.

### Criteria for discontinuing or modifying allocated interventions

If the patient experiences any drug-related safety issues (such as severe bleeding, an unexplained decline in hemoglobin, clinically significant coagulation abnormalities, severe hypotension, or an allergic reaction to the study drug), the use of the assigned anticoagulant will be discontinued or adjusted. If appropriate dose adjustments are made but recurrent circuit coagulation still occurs, or if the attending physician determines that the assigned anticoagulant is no longer appropriate for the patient’s condition, further modification of the intervention will be considered. All changes and their corresponding reasons will be documented.

### Strategies to improve adherence to interventions

This intervention will be implemented by trained research staff during the continuous renal replacement therapy (CRRT) process of the patients. The patients are unaware of the intervention measures they are receiving and will not be involved in the medication administration process. The researchers will monitor the patients’ compliance through bedside checklists and daily review of treatment records. Any deviations from the trial protocol will be recorded along with the reasons.

### Relevant concomitant care permitted or prohibited during the trial

The standard treatment measures for severe acute pancreatitis are permitted. As long as they meet the clinical indications, any drugs that do not interfere with continuous renal replacement therapy (CRRT) or designated anticoagulation treatment can be used. Except for the specified protocols, the use of additional systemic anticoagulants is prohibited. Hemoperfusion, combined CRRT–hemoperfusion, or other extracorporeal cytokine adsorption therapies will not be permitted during the intervention period because they may independently alter inflammatory biomarker concentrations and confound assessment of study outcomes. All concomitant treatments and any deviations from the protocol will be recorded.

### Provisions for post-trial care

The potential risks of this study are mainly related to the anticoagulation treatment during continuous renal replacement therapy, which may increase the risk of coagulation abnormalities. After the trial is completed, the participants will continue to receive standard treatment for severe acute pancreatitis. Throughout the entire study period, adverse events will be closely monitored. If any adverse events occur, appropriate medical intervention will be provided promptly.

### Outcomes

The primary endpoint of this study is the average service life of the CRRT circuit or filter. The functional lifespan of CRRT circuit begins when the extracorporeal circulation of blood starts and ends when the circuit is taken out of service by intensive care unit (ICU) staff (when the treatment goal is achieved, death occurs, or the upper limit of 72 h for filters). The reasons for ICU staff to stop the circuit operation included: (1) the transmembrane pressure on both sides of the circuit exceeded 300 mmHg, (2) visible blood clots hindered the blood flow within the machine, (3) the blood pump could not rotate due to blood clot blockage, (4) other (recorded by the bedside staff). The first three reasons are considered to be cases of circuit clotting. If the bedside staff recorded “other” or the reason for stopping the circuit was missing, adjudication is required. Two senior critical care specialists, who were not involved in the research, independently reviewed these cases and adjudicated the reasons for circuit discontinuation. In case of any disagreement, they resolved it through joint consultation. The outcome of the judgment may be one of three types (coagulated, non-coagulated or unknown condition). The circuit that is selectively discontinued due to reasons during the treatment process (such as transfer within the hospital) is regarded as “non-coagulated.”

The secondary endpoints of this study include: 1. SOFA and APACHE II scores were evaluated on days 1, 3, 5, and 7 after enrollment, and the changes from the baseline (ΔSOFA /ΔAPACHE II) were calculated to reflect the trend of disease progression; 2. Serum levels of pancreatic enzymes (amylase and lipase) and PCT, CRP, IL-6, IL-8, IL-10 and TNF-α were measured on days 1, 3, 5, and 7, and their changes from baseline were analyzed; 3. levels of coagulation parameters(include aPTT, PT, fibrinogen, platelet count) were assessed on specific days (day 1, 3, 5, and 7) together with their changes from baseline values; 4. time to successful initiation of enteral nutrition; 5. blood transfusion requirements(RBC, FFP, platelet units), 6. ICU length of stay and total hospitalization days; 7. Clinical outcomes (blood purification dependency rate, length of ventilator use and all-cause mortality at 28 days).

Safety outcomes were defined as the occurrence of any adverse events (AEs), including serious adverse events (SAEs) and adverse drug reactions (ADRs), such as the occurrence of any bleeding events (as defined by the International Society on Thrombosis and Haemostasis), allergic reactions, and heparin-induced thrombocytopenia. Researchers are required to continuously monitor for potential adverse reactions throughout the study. In the event of any adverse event, the researchers will promptly take appropriate measures to ensure the safety of the subjects and keep detailed records. For serious adverse events, researchers are required to report the incidents to the ethics committee within 24 h. Study termination will be considered according to the severity and nature of the event.

### Participant timeline

Participants will be followed for 28 days. At the final visit of the last participant, clinical status data of all individuals, regardless of trial completion or withdrawal, will be obtained. The details of the timeline are presented in Table 1.

**TABLE 1:** Schedule of enrolment, interventions, and assessments.

Timepoint	Study period					
	Enrolment	Allocation	Intervention	Days 3, 5, 7	CRRT period	Day 28/discharge
Timepoint	-t1	0	t1	t2∼t5		t6
Enrollment: Eligibility assessment Informed consent Allocation Etiology, baseline radiological severity (CTSI/EPIC score), IAH grade, surgical/interventional procedures						
	X					
	X					
					
	X					
Interventions: Nafamostat mesylate Heparin						
		X	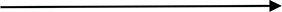		X	
		X	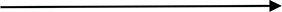		X	
Assessments: CRRT circuit/filter lifespan SOFA and APACHE II scores§ Inflammatory biomarkers* Pancreatic enzymes (amylase, lipase)* Coagulation parameters† Time to successful enteral nutrition Blood transfusion requirements ICU and hospital length of stay Clinical outcomes‡ Adverse event monitoring End-of-study assessment						
			X	X	X	
		X		X		
		X		X		
		X		X		
		X		X		
				X		
				X		
						X
						X
			X	X	X	X
						X

*Inflammatory biomarkers and pancreatic enzymes include PCT, CRP, IL-6, IL-8, IL-10, and TNF-α, amylase, and lipase, assessed at baseline (Allocation, Day 1) and on days 3, 5, and 7 after enrollment. †Coagulation parameters include aPTT, PT, fibrinogen, and platelet count, assessed at baseline (Allocation, Day 1) and on days 3, 5, and 7 after enrollment. ‡Clinical outcomes include blood purification dependency, duration of mechanical ventilation, and all-cause mortality at 28 days. §SOFA and APACHE II scores are assessed at baseline (Allocation, Day 1) and on days 3, 5, and 7 after enrollment.

### Sample size estimation

The sample size was estimated based on the primary endpoint, which was average service life of the CRRT circuit lifespan or filter (hours). The assumed baseline value of approximately 30 h for the heparin group, together with the standard deviation used in this calculation, were derived from the pooled filter-lifespan data summarized in the network meta-analysis of CRRT anticoagulation strategies by Zhou et al. and the review of circuit-survival determinants by Tsujimoto and Fujii, which indicate that heparin-anticoagulated circuits typically survive on the order of 20–40 h with considerable between-study variability (17, 32). Because the standard deviation of filter lifespan varies significantly across studies, we deliberately chose a relatively conservative value (σ = 8 h) toward the higher end of the reported range, to avoid underestimating the required sample size. A minimum clinically important difference of 4 h (an approximately 13% relative extension over the assumed heparin baseline) was prespecified not purely on statistical grounds but because a gain of this magnitude would be expected to translate into approximately one fewer circuit change per patient over a typical treatment course, with corresponding reductions in nursing workload and blood loss associated with circuit priming and disconnection, as well as lower filter and consumable costs—benefits that are clinically and economically meaningful even though the absolute increase in hours is modest. Assuming a two-sided α level of 0.05 and a power (1–β) of 80%, the sample size was estimated using the approximate formula for comparing the means of two independent samples, resulting in a sample size of approximately 63 cases in each group. Taking into account the approximately 10% dropout rate, it is necessary to recruit 70 participants in each group.

### Recruitment

The recruitment of participants was conducted in the intensive care unit of Weifang People’s Hospital. This intensive care unit has 110 beds. The researchers will conduct a comprehensive assessment of admitted patients. If the patients meet the inclusion criteria, the researchers will fully explain the study to the patients or their legal representatives and answer all their questions, ultimately assisting in completing the informed consent procedure.

### Assignment of interventions: allocation

#### Sequence generation

A statistician who was not involved in the trial used the SPSS 24.0 software to generate a random allocation sequence. Based on the random allocation results, the participants were randomly assigned to the nafamostat mesylate group or the heparin group in a 1:1 ratio.

#### Allocation concealment mechanism

The randomized code and allocation results will be encapsulated in an opaque envelope and handed over to designated personnel for proper custody. To ensure the fairness of the evaluation of the trial results, the randomized results will not be known to the personnel involved in data collection and statistics.

#### Implementation

After obtaining the informed consent of the participants, the trained researchers will open the corresponding envelopes. Participants were randomly assigned to the corresponding groups based on the results in the envelopes (the nafamostat mesylate group or the heparin group).

### Assignment of interventions: blinding

#### Who will be blinded

Due to the differences in management methods and monitoring requirements for various intervention measures, it is not feasible to implement blinding for clinical physicians and nurses. Therefore, this trial will be an open-label trial, but the result evaluators and statisticians will not know the specific treatment allocation.

#### Procedure for unblinding if needed

Not applicable. No blinding was implemented for participants or clinicians. Outcome assessors and statisticians were blinded to group allocation. Therefore, no unblinding procedure was required.

### Data collection and management

#### Plans for assessment and collection of outcomes

We collect data using case report forms. When patients are enrolled in the study, researchers will collect relevant demographic data [such as gender, age, and body mass index (BMI)] from the hospital’s medical records, major comorbidities (such as hypertension, diabetes, coronary artery disease, etc.), the causes of pancreatitis, and use the SOFA and APACHE II scores to assess the severity of the patients’ conditions. To further characterize baseline disease severity, the etiology of pancreatitis (biliary, alcoholic, hypertriglyceridemia-related, post-ERCP, or other), hemodynamic status at enrollment (mean arterial pressure, vasopressor requirement and dose, and serum lactate), radiological severity on the earliest available abdominal CT scan scored using the CT Severity Index (CTSI) and the Extrapancreatic Inflammation on CT (EPIC) score (23), which quantifies pleural effusion, ascites, and retroperitoneal or mesenteric fluid collections, the presence and grade of intra-abdominal hypertension (IAH, defined by bladder pressure measurement according to World Society of the Abdominal Compartment Syndrome criteria), and any surgical or interventional procedures performed (e.g., percutaneous catheter drainage, minimally invasive or open necrosectomy, decompressive laparotomy) will also be recorded at baseline.

Furthermore, the researchers will collect the necessary data based on the results of the laboratory tests, and record the therapeutic measures taken, the disease progression, as well as all adverse events (AE) observed. Laboratory data will comprise blood culture results, white blood cell count, liver and renal function parameters, and coagulation profiles. Disease progression will be assessed using baseline severity scores and serum biomarkers, including the Sequential Organ Failure Assessment (SOFA) score, the Acute Physiology and Chronic Health Evaluation II (APACHE II) score, serum pancreatic enzymes (amylase and lipase), procalcitonin (PCT), C-reactive protein (CRP), interleukin-6 (IL-6), interleukin-8 (IL-8), interleukin-10 (IL-10), and tumor necrosis factor-α (TNF-α). On days 1, 3, 5, and 7 after randomization, follow-up data will be collected, and changes from baseline will be derived to examine longitudinal trends.

#### Plans to promote participant retention and complete follow-up

This study will be conducted in the ICU, and most outcomes will be assessed during hospitalization. Therefore, the risk of loss to follow-up is expected to be very low. Monitoring during CRRT and standard clinical and laboratory assessments are part of routine intensive care unit care. If the patient is transferred to general ward before all planned observations are completed, the required result data will be obtained from the electronic medical record. If the patient has been discharged, the researchers will contact the participant or their family by phone. Reasons for withdrawal or incomplete follow-up will be recorded.

#### Data management

We have established an independent data management team. The members of this team were unaware of the treatment allocation. They were responsible for regular data audits and source data verification. In addition, this independent data management team was also responsible for the statistical analysis of the final data. To protect patient privacy, the personal identification information of the patients was removed and replaced with the study number before analysis. The data management team will conduct strict supervision over the data. The ethics committee has the right to terminate this research under reasonable circumstances.

#### Confidentiality

The personal information of all participants will be replaced by unique identification numbers. Access to the data will be restricted to authorized members of the data management team. All data will be securely stored.

#### Plans for collection, laboratory evaluation and storage of biological specimens for genetic or molecular analysis in this trial/future use

Not applicable. This study will not involve the collection, storage, or analysis of biological samples.

### Statistical methods

#### Statistical methods for primary and secondary outcomes

Continuous variables were tested for normality using the Kolmogorov–Smirnov test. Normally distributed data were presented as mean ± standard deviation (SD) and compared using the independent-samples *t*-test. Non-normally distributed variables were reported as median (interquartile range, IQR) and analyzed using the Mann–Whitney U test. Categorical variables were expressed as counts (percentages) and compared using the χ^2^ test or Fisher’s exact test, as appropriate.

In addition, the primary endpoint of this study was the mean lifespan of the hemodialysis filter during CRRT. Kaplan–Meier survival analysis was conducted to compare the cumulative probability of filter life over time, with the log-rank test applied for group comparison. To control for potential confounders (e.g., disease severity scores), multivariable linear regression and Cox proportional hazards models were used as sensitivity analyses.

Secondary outcomes included the changes in disease severity scores (SOFA and APACHE II), pancreatic enzymes (amylase, lipase) and inflammatory biomarkers (PCT, CRP, IL-6, IL-10, TNF-α) measured on Day 1 (baseline), day 3, day 5, and day 7 after randomization. To evaluate time-dependent trends and group differences, a repeated-measures analysis of variance (RM-ANOVA) was applied for normally distributed variables. Baseline disease severity characteristics, including etiology, CTSI/EPIC radiological scores, and the presence and grade of intra-abdominal hypertension, will be summarized descriptively and compared between groups using the χ^2^ test, Fisher’s exact test, or the Mann–Whitney U test, as appropriate. The assumption of sphericity was tested by Mauchly’s test, and when violated, the Greenhouse–Geisser correction was used to adjust degrees of freedom. Differences in change-from-baseline values (ΔSOFA, ΔAPACHE II, and Δbiomarker levels) between groups were analyzed using the independent-samples *t*-test or Mann–Whitney U test, as appropriate. Correlations between the changes in inflammatory markers and changes in severity scores were assessed using Spearman’s rank correlation coefficient.

All participants who received at least one dose of study anticoagulant were included in the safety set. The incidence of adverse events (AEs), serious adverse events (SAEs), and bleeding or coagulation complications were summarized using descriptive statistics and compared between groups with the chi-square test or Fisher’s exact test, as appropriate.

All statistical analyses were performed using SPSS version 26.0 (IBM Corp., Armonk, NY, United States) and R version 4.3.0 (R Foundation for Statistical Computing, Vienna, Austria). A two-sided *p*-value < 0.05 was considered statistically significant.

#### Interim analyses

No formal interim analysis of treatment efficacy is planned, given the short treatment and follow-up duration of this trial. However, independent of any efficacy assessment, a pre-specified safety stopping rule has been established. The DMC will conduct an ad hoc safety review whenever any of the following occurs in either study arm: (1) the incidence of major bleeding (defined by ISTH criteria) reaches or exceeds 20% of enrolled participants in that arm; (2) two or more cases of heparin-induced thrombocytopenia (in the UFH arm) or two or more cases of severe anaphylactoid/hyperkalemic reactions attributable to nafamostat mesylate (in the NM arm) are reported; or (3) any single adverse event is judged by the treating physician and confirmed by the DMC to be directly and probably related to the study anticoagulant and life-threatening. Upon triggering any of these thresholds, the DMC will unblind and review the relevant safety data and may recommend continuation without modification, protocol amendment (e.g., dose adjustment or revised monitoring), or early termination of the trial to the trial steering committee. These numerical thresholds will be reviewed and, if necessary, refined by the DMC biostatistician prior to trial initiation.

#### Methods for additional analyses (e.g., subgroup analyses)

Subgroup analyses will assess whether treatment effects differ across predefined etiologies of acute pancreatitis (biliary, alcoholic, hypertriglyceridemia-related, post-ERCP, and other). Adjusted analyses will be performed using multivariable regression models to control for potential confounding factors.

#### Methods in analysis to handle protocol non-adherence and any statistical methods to handle missing data

Because of the prospective design and short follow-up period of this trial, the occurrence of missing data in this study will be rare. Protocol deviations will be incorporated within an intention-to-treat framework, supported by a per-protocol analysis, while missing data will be handled primarily with mixed-effects models and, when necessary, multiple imputation with sensitivity checks.

#### Plans to give access to the full protocol, participant level-data and statistical code

Requests for the full protocol, participant-level data, and related analysis code will be considered by the corresponding author on reasonable request.

### Oversight and monitoring

#### Composition of the coordinating center and trial steering committee

The entire trial will be coordinated by the ICU of Weifang People’s Hospital. The trial steering committee consists of senior researchers, independent clinicians, statisticians and research coordinators. They are responsible for providing overall guidance, monitoring safety and offering suggestions for the protocol amendments.

#### Composition of the data monitoring committee, its role and reporting structure

An independent data monitoring committee (DMC) will oversee the safety and integrity of the trial. This committee will consist of experienced clinicians, an ethics committee secretary, a director of the intensive care unit, and a statistician. These personnel all come from Weifang People’s Hospital. DMC will regularly review the safety data and adverse events, and discuss any new potential risks that may arise, guided by the pre-specified safety stopping thresholds described in the Interim analyses section {21b}. The discussion results and related suggestions will be presented in the form of a report to the trial steering committee.

#### Adverse event reporting and harms

From the moment patient is enrolled, all adverse events will be closely monitored. The main adverse events of this study are related to changes in coagulation function. When an adverse event occurs, the treating physician will immediately provide appropriate medical management to ensure participant safety. All adverse events will be promptly reported to the study coordinator and to the ethics committee within 24 h. Each adverse event will be detailedly recorded in the case report form. All adverse events will be summarized in the final publication.

#### Frequency and plans for auditing trial conduct

The Ethics Committee of Weifang People’s Hospital will oversee adherence to ethical standards throughout the study. The Research Committee will review participant recruitment, informed consent procedures, protocol compliance, and recorded adverse events at 6-month intervals.

#### Plans for communicating important protocol amendments to relevant parties (e.g., trial participants, ethical committees)

The study protocol has been approved by the Ethics Committee of Weifang People’s Hospital. Any modification to the agreement must be reviewed and approved by the Ethics Committee of Weifang People’s Hospital, and the information must be updated on the Chinese Clinical Trial Registry.

#### Dissemination plans

Study results will be presented at relevant national and international conferences and submitted for publication in peer-reviewed journals.

## Discussion

Severe acute pancreatitis can lead to multiple organ dysfunction and is closely associated with a high mortality rate (1, 24). Standardized treatment for patients with severe acute pancreatitis is of great significance in improving their prognosis. The treatment of severe acute pancreatitis in the intensive care unit is mainly supportive, based on fluid infusion, enteral nutrition, organ support and anti-infection therapy. Continuous renal replacement therapy (CRRT) CRRT is a type of treatment in hemodialysis. It can alleviate the inflammatory response of the body by removing inflammatory mediators (25–27). Previous studies (28, 29) have shown that CRRT is beneficial for patients with SAP. Early initiation of CRRT can improve the prognosis of patients with SAP (30). A recent single-center retrospective study by Saullo et al. further supports this rationale (31). Due to the clotting phenomenon within the blood circuit (including the blood filter), the efficacy of CRRT will be reduced and it may lead to blood loss as well as a decrease in solute clearance rate. Therefore, an important prerequisite for the smooth progress of continuous renal replacement therapy (CRRT) is to maintain the unobstructed circulation of the pipeline (32). External blood anticoagulation is one of the strategies for achieving the goal of unobstructed and continuous circulation of the pipeline.

In clinical practice, the most commonly used anticoagulant in CRRT is heparin. Additionally, studies (14, 33) have shown that heparin can improve the microcirculation of the pancreas during severe acute pancreatitis (SAP) and reduce the release of inflammatory mediators. However, heparin may increase the risk of bleeding, especially in patients with coagulation disorders, gastrointestinal bleeding, or SAP. Because patients with severe acute pancreatitis often have difficulty maintaining a good balance between a systemic hypercoagulable state and the risk of bleeding. Nafamostat mesylate is a synthetic serine protease inhibitor (34). Due to its potent anticoagulant activity, it is used in the treatment of extracorporeal circulation anticoagulation in blood purification. The half-life of nafamostat mesylate is short, and it has a relatively minor impact on the body’s coagulation function (34–36). What is important is that nafamostat mesylate can also inhibit complement activation and cytokine release, thereby reducing the inflammatory response (37, 38). Therefore, nafamostat mesylate is widely used in the treatment of acute pancreatitis. However, nafamostat mesylate also carries risks such as bleeding, thrombocytopenia, and electrolyte imbalance. So far, in the process of the CRRT for patients with SAP, the efficacy and safety of nafamostat mesylate and heparin as anticoagulants remain unclear.

We designed a randomized controlled trial, with the average filter lifespan as the primary outcome measure, aiming to compare the anticoagulant effects of nafamostat mesylate and heparin during CRRT treatment in patients with SAP. Secondary outcomes, including changes in SOFA scores, APACHE II scores, pancreatic enzymes (amylase, lipase), and serial inflammatory biomarkers (PCT, CRP, IL-6, IL-8, IL-10, TNF-α), will help elucidate the anti-inflammatory effects of nafamostat mesylate beyond its anticoagulant role. To reduce methodological heterogeneity, the CRRT modality has been standardized as continuous venovenous hemodiafiltration (CVVHDF) for all enrolled participants because circuit lifespan may differ across CRRT modalities. In addition, concomitant hemoperfusion or other extracorporeal cytokine adsorption therapies will not be permitted during the intervention period to minimize confounding of both the primary endpoint and inflammatory biomarker analyses. In addition, we will also record the occurrence of adverse events and compare the safety of using nafamostat mesylate versus heparin anticoagulation in CRRT treatment for patients with SAP. This protocol focuses on circulating biomarker dynamics as a marker of the systemic inflammatory response; due to resource constraints, direct measurement of cytokine concentrations in the CRRT effluent was not incorporated into the current design, but represents a valuable direction for future mechanistic studies of the anti-inflammatory effect of nafamostat mesylate during blood purification (31). In summary, this study aims to compare the anticoagulant efficacy and safety differences between nafamostat mesylate and heparin in patients with SAP who receive CRRT. The results of this study are expected to provide guidance for the selection of anticoagulant drugs for critically ill patients with SAP who receive CRRT, and to clarify the optimal anticoagulation strategy for this population, thereby optimizing clinical practice for treating SAP patients.

### Strengths and limitations of this study

This trial has several limitations. First, because it is not feasible to blind bedside clinicians and nurses to the anticoagulant used during CRRT, the trial is open-label, and the primary outcome of filter lifespan is partly determined by bedside staff decisions about when to discontinue the circuit. Even though outcome assessors, adjudicators, and the statistician remain blinded to treatment allocation, awareness of the assigned anticoagulant could subtly influence the threshold at which bedside staff choose to stop a circuit, introducing a theoretical source of bias that blinding of assessment alone cannot fully eliminate. To minimize this risk, we have prespecified objective, mechanically verifiable stopping criteria (transmembrane pressure exceeding 300 mmHg, visible clotting impeding blood flow, or pump failure due to clot obstruction) as the primary safeguard against subjective variation in circuit-discontinuation decisions, and cases in which the recorded reason is missing or classified as “other” are independently adjudicated by two blinded senior intensivists, with disagreements resolved by consensus. Nonetheless, residual detection bias related to the open-label design cannot be entirely excluded and should be considered when interpreting the primary outcome. Second, as a single-center trial, the generalizability of our findings to other institutions and case-mix populations may be limited, and multicenter validation will be needed. Third, resource constraints precluded direct measurement of cytokine clearance across the filter itself, so the anti-inflammatory effect of nafamostat mesylate is inferred from circulating biomarker trends rather than effluent-based clearance data.
